# Development of Healthier and Functional Dry Fermented Sausages: Present and Future

**DOI:** 10.3390/foods11081128

**Published:** 2022-04-14

**Authors:** Noelí Sirini, Paulo E. S. Munekata, José M. Lorenzo, María Ángeles Stegmayer, Mirian Pateiro, José Ángel Pérez-Álvarez, Néstor Sepúlveda, María Elena Sosa-Morales, Alfredo Teixeira, Juana Fernández-López, Laureano Frizzo, Marcelo Rosmini

**Affiliations:** 1 Laboratory of Food Analysis “Med. Vet R. Dalla Santina”, Institute of Veterinary Science (ICiVet Litoral), National University of the Litoral—National Council of Scientific and Technical Research (UNL/CONICET), Esperanza 3080, Province of Santa Fe, Argentina; noesirini@gmail.com (N.S.); angie.stegmayet@gmail.com (M.Á.S.); lfrizzo@fcv.unl.edu.ar (L.F.); 2 Centro Tecnológico de la Carne de Galicia, Avd. Galicia No. 4, Parque Tecnológico de Galicia, San Cibrao das Viñas, 32900 Ourense, Spain; jmlorenzo@ceteca.net (J.M.L.); mirianpateiro@ceteca.net (M.P.); 3 Universidade de Vigo, Área de Tecnoloxía dos Alimentos, Facultade de Ciencias, 32004 Ourense, Spain; 4 IPOA Research Group (UMH-1 Grupo REVIV-Generalitat Valenciana), Dept Tecnología Agroalimentaria, Escuela Politécnica Superior de Orihuela, Universidad Miguel Hernández, Ctra Beniel, Km 3.2, 03312 Orihuela, Spain; ja.perez@umh.es (J.Á.P.-Á.); j.fernandez@umh.es (J.F.-L.); 5 Centro de Tecnología e Innovación de la Carne (CTI-Carne), Universidad de La Frontera, Temuco 4780000, Chile; nestor.sepulveda@ufrontera.cl; 6 Departamento de Producción Agropecuaria, Facultad de Ciencias Agropecuarias y Forestales, Universidad de La Frontera, Temuco 4780000, Chile; 7 Departamento de Alimentos, División de Ciencias de la Vida, Campus Irapuato-Salamanca, Universidad de Guanajuato, Irapuato 36500, Guanajuato, Mexico; msosa@ugto.mx; 8 Escola Superior Agrária, Instituto Politécnico de Bragança, Apartado 172, 5301-855 Bragança, Portugal; teixeira@ipb.pt; 9 Department of Public Health, Faculty of Veterinary Science, National University of the Litoral, Esperanza 3080, Province of Santa Fe, Argentina

**Keywords:** functional ingredients, sodium chloride, saturated fat, dietary fiber, probiotics, postbiotics, nitrite, consumer preference

## Abstract

In recent years, consumer perception about the healthiness of meat products has changed. In this scenario, the meat industry and the scientific and technological areas have put their efforts into improving meat products and achieving healthier and functional formulations that meet the demands of today’s market and consumers. This article aims to review the current functional fermented meat products, especially on sausage development. Firstly, an emphasis is given to reducing and replacing traditional ingredients associated with increased risk to consumer’s health (sodium, fat, and nitrites), adding functional components (prebiotics, probiotics, symbiotics, and polyphenols), and inducing health benefits. Secondly, a look at future fermented sausages is provided by mentioning emerging strategies to produce innovative healthier and functional meat products. Additional recommendations were also included to assist researchers in further development of healthier and functional sausages.

## 1. Introduction

Recent epidemiological studies have documented that meat product consumption is related to the prevalence of a wide range of emerging diseases like obesity, cancer, heart-related diseases, and various other disorders [1,2]. In the 21st century, since the focus of nutrition is shifting toward the concept of “optimal nutrition”, reformulation or addition of functional products have acquired the interest of researchers and professionals as successful strategies to fulfil consumer perceptions of current food healthiness [3]. In this context, the interest towards healthier and functional foods from consumers has intensively increased, and its development appears to be a long-term trend with important market potential of 10 billion dollars in the period of 2021–2030 [4].

The term “functional food” was created in Japan in 1984. It was defined as “food containing an ingredient with functions for health and officially approved to claim their physiological effects on the human body”. However, healthier and functional foods have no universally accepted definitions and many authors have raised a discussion about the proper definition of this food category [5,6].

The technological development of meat products to meet this demand is a challenging task that involves supportive information from related areas and the preservation or improvement of quality, safety, and shelf life of meat products [7]. Reducing or replacing an ingredient associated with increased risk of developing a disease is centered in the current and known formulation of meat products. In this context, sodium chloride, nitrate and nitrite salts, and saturated fat are important ingredients that have been receiving great attention from researcher in recent decades [8]. In the case of functional meat products, the development consists of the addition of components that may exert a beneficial health effect such as prebiotics, probiotics, symbiotics, and polyphenols [9,10].

**Figure 1:**
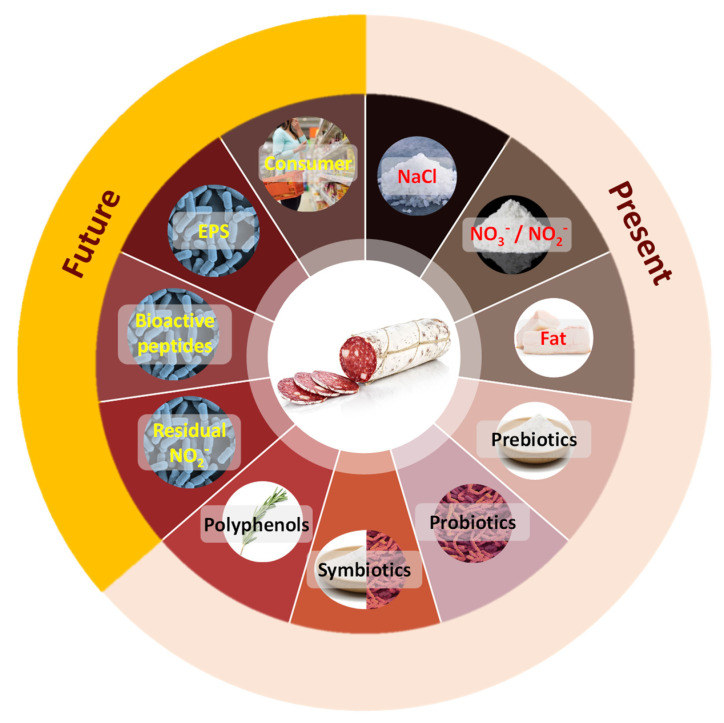
Main factors in the development of healthier and functional fermented meat products.

This current demand for innovative and healthy food products represents a stimulus for the development of new fermented meat products [11]. Therefore, food technologists are currently focusing on developing meat and meat products with benefits to promote health and reduce the risk of some diseases by replacing specific components or by incorporating bioactive components [12]. This review aims to provide an overview of the current and emerging lines of research to produce healthier and functional dry fermented sausages (Figure 1) in two sections. The first section discusses current and widely studied strategies to produce healthier and functional meat products. The second section is composed of emerging and promising strategies to be explored in the upcoming years.

## 2. Present Healthier and Functional Fermented Meat Products

### 2.1. Reduction or Replacement of Ingredients of Major Concern for Consumers

#### 2.1.1. Sodium Chloride

Sodium chloride is an essential multi-functional ingredient in dry fermented sausages. Sodium chloride is involved in myofibrillar protein solubilization, enhancing water binding and water retention capacity (thus resulting in the formation of a desirable gel texture), decreasing water activity (a_w_; thus controlling the growth of pathogen microorganisms), promoting characteristic sensory attributes, and finally, controlling biochemical and enzymatic reactions during ripening [13,14]. However, the global daily sodium intake, estimated at 9–12 g, is double the recommended maximum levels [15,16]. A diet high in sodium increases the risk of developing cardiovascular diseases (especially hypertension) and it is also considered to be a risk factor for other diseases (such as obesity, kidney stones, and some cancers) [17]. Thus, the World Health Organization established the reduction of 30% content in salt and sodium intake as a global target for 2025 with the aim of progressively adapting consumers to new and less salty foods [18], particularly for meat products, in which the content can achieve 5 g/100 g [19,20].

Therefore, reducing sodium content in fermented meat products may be useful to reduce the daily sodium intake for meat products consumers. However, it is important to note that it represents a major challenge for the meat industry, since sodium reduction can cause a loss of quality and also lead to safety concerns. A low sodium content could also affect flavor and taste due to protein oxidation, lipid oxidation, and lipolysis, which are the most remarkable characteristics and important indexes for quality evaluation [21]. Sodium reduction in dry fermented sausages formulation could increase the activity of proteolytic enzymes, resulting in a softer and more brittle texture due to the higher degradation of myofibrillar proteins [21]. Furthermore, the inhibition of pathogenic microorganisms could not be guaranteed.

Nowadays, in order to reduce health risks, many authors have studied the effects of sodium reduction as well as different sodium substitutes in technological properties and bacterial counts in fermented sausages. One example is the study of NaCl content on protein oxidation, lipid hydrolysis, and oxidation during the processing of Chinese dry sausage by Zhao et al. [22]. Two groups of Chinese dry sausages with 2% and 4% salt content were examined. The researchers concluded that NaCl curing salt content had significant effects on protein oxidation, lipid oxidation, and lipolysis in the Chinese dry sausages production, and they assert that 4% NaCl could facilitate protein oxidation, lipid hydrolysis, and oxidation compared to 2% NaCl treatment. The effect of NaCl on lipase activity was shown, probably because of the high content of NaCl that accelerated fatty acid oxidation. Corral et al. [23] have also reported NaCl as a pro-oxidant, since it can promote oxidation by disrupting the structural integrity of the cell membrane and facilitating oxidative reactions between oxidizing agents and unsaturated lipids in meat products. On the other hand, the pro-oxidant effect of NaCl in fermented sausages was not reported by Hu et al. [24], with NaCl levels between 1.0% and 2.5%. This could be explained by both authors having used different amounts of NaCl in their fermented sausages.

The flavor in dry sausages is mainly derived from lipid oxidation and hydrolysis, changes of neutral lipids, free fatty acids, and phosphatides. The reduction of NaCl concentration could produce an increase in the activity of endogenous enzymes (such as lipases) [21]. Consequently, the addition of sodium could represent product-dependent variable that is desirable in terms of physicochemical characteristics and sensory attributes (Table 1). It is known that a satisfactory flavor during dry fermented sausage processing is achieved by moderate lipid oxidation and hydrolysis [21]. However, excessive oxidation may bring rancidity, a nauseous smell, and an unpleasant flavor. Additionally, it could also affect consumers’ health. A sensorial evaluation is recommended in case of NaCl reduction or replacement. In this context, Hu et al. [24] found that a higher overall acceptability was found in sausages with 2.0% NaCl in relation to sausages produced with 4.0% NaCl. Hence, the authors finally reported that 2.0% NaCl can be employed as the right level of addition in dry fermented sausage by achieving better quality and flavor with no effects in physicochemical and sensory properties.

Additionally, Hu et al. [24] evaluated the effects of NaCl reduction from 2.5% to 1.0% (2.5%, 2.0%, 1.5%, and 1.0%) on the growth of starter cultures during the production of dry fermented sausages. As a result, the highest counts of *Staphylococcus* spp. were observed in sausages with 2.0% and 2.5% NaCl, since the NaCl proportion increased along the fermented period due to water loss, thus favoring *Staphylococcus* spp. growth. In the case of lactic acid bacteria (LAB), increasing NaCl content to 2.5% caused a reduction in the counts of these bacteria. Although some variation is associated with NaCl level of starter cultures, both bacteria successfully dominated the sausage mass and promoted the development of expected characteristics of dry fermented sausage.

Replacing NaCl by other chloride salts is another relevant strategy to reduce sodium content in dry fermented sausages. Although NaCl cannot be completely replaced by other sodium substitutes, the partial replacement with KCl has been indicated as an interesting solution [31,32]. However, a bitter and metallic taste has been perceived in products with high KCl replacement (30–40%); in addition, high KCl levels in foods could increase the risk of suffering several illnesses like diabetes, adrenal insufficiency, and chronic kidney disease [33]. Glutamate, amino acids, and citric acid are usually used as flavor enhancers and as sodium substitutes to mask the defects of KCl [31,32]. Additionally, Corral, Salvador, and Flores [23] suggested that, in order to improve the aroma of reduced salt of fermented sausages, it is necessary to search for other alternatives to KCl in dry fermented sausage with reduced NaCl content.

In this context, Chen et al. [25] studied the flavor profile of dry fermented sausage with different NaCl substitutes by using headspace solid phase micro-extraction gas chromatography-mass spectrometry (HS-SPME-GC-MS) combined with electronic nose and electronic tongue. The data from electronic tongue assay and sensory evaluation showed that the taste profile of the 70% NaCl, 20% KCl, and 10% flavor enhancer (4% lysine, 3.5% maltodextrin, 1% alanine, 1% calcium lactate, and 0.5% citric acid) treatment was similar to the control treatment (100% NaCl). In addition, the substitution of 30% of NaCl with 20% KCl and 10% flavor enhancers favored the accumulation of moisture and LAB growth, reduced the pH, and limited *Staphylococcus* growth compared to other treatments. These results showed that an ideal low-sodium substitute was achieved with a 30% reduction in NaCl.

#### 2.1.2. Nitrate and Nitrite Salts

The use of nitrates and nitrite salts in dry-cured meat products has technological and safety benefits. These compounds inhibit pathogenic bacteria growth (especially *Clostridium botulinum*), delay oxidative rancidity, and contribute to the development of the typical cured meat flavor and color [34]. However, the consumption of nitrosamines (a product from the reaction between nitric oxide and secondary amines) can increase the risk of developing cancer [35]. The use of nitrate and nitrite salts in the meat industry is strictly regulated because of their acute toxicity [36].

Nowadays, one of the most relevant strategies to change the negative image of cured meat products is the improvement of their nutritional value by adding organic and natural components [37]. The use of natural extracts rich in nitrates (commonly as a lyophilized product) could accomplish both technological requirements (physicochemical, microbiological, and sensory characteristics) and consumer demand (removal of E-number from the product by getting a clean label) [38,39]. Although at the moment there is no exact regulatory definition for the term ‘clean label’, it is known that foods should not contain artificial colorants and additives and could have reduced levels of synthetic additives [40].

In cured meat products, clean label claims could be achieved by substituting commercial nitrites with the use of vegetable extracts with a high nitrate content in their composition. In addition, there are many specific bacterial starter cultures, such as *Staphylococcus carnosus* [41], that have the ability to reduce nitrates (from vegetable sources) to nitrites and to develop cured characteristics in meat products [42]. There are two different strategies to use vegetable extracts in meat processing: the endogenous formation of nitrite (during initial maceration) and the use of a pre-fermented vegetable extract for the intentional conversion of nitrate to nitrite [39]. In the case of fermented sausages, the use of nitrate-rich extracts is the most common strategy.

Different sources have been proposed (spinach, celery, radishes, and beetroots) for the elaboration of fermented meat products due to their high nitrate contents [43,44]. Additionally, in the case of radishes and beetroots, these natural sources of nitrate could also contribute to the development of color in meat products. In this regard, Ozaki et al. [26] studied the presence of beetroot and radish powders as a natural nitrites source for dry fermented sausages. Treatments with different amounts of radish powder (0.5% and 1%) and beetroot powder (0.5% and 1%) were evaluated during the ripening process and storage time. During ripening process, nitrites were formed from radish and beetroot powders. Additionally, the addition of radish and beetroot powders favored the decrease of a_w_ by contributing to the safety of the final product. The natural extracts also favored the decrease of moisture and an increment in the weight loss of sausages. Regarding microbiological analyses, all treatments after 35 days of drying and throughout the 60 days of storage could be considered safe for consumption. TBARS results indicate radish powder delayed lipid oxidation in fermented dry sausages. However, 1% beetroot powder treatments showed TBARS values above the limit for sensory perception of rancidity (2 mg/kg) after 60 days of storage. Additionally, beetroot powder’s addition to sausages negatively affected the color due to the presence of betalains (natural pigments responsible for the characteristic purple color of beetroot). Finally, the 1% radish powder treatment was suggested as a potential nitrite replacer obtained from a simple and feasible drying process.

Another line of thought considered is the use of natural extracts rich in compounds with antioxidant and antimicrobial activity. For instance, Aquilani et al. [27] studied the feasibility of producing a dry fermented sausage by replacing sodium nitrite with natural extracts. The dry fermented sausages were manufactured replacing sodium nitrite with two mixtures of natural extracts consisting of grape seed extract and olive pomace hydroxytyrosol (GSE), and chestnut extract and olive pomace hydroxytyrosol (CHE). Plant extract as a replacement for nitrites represent a good alternative since plant extracts are very rich in polyphenols, flavonoids, and terpenoids, and are able to perform the required double antioxidant-antimicrobial functions [45]. Additionally, GSE and CHE mixtures showed a slightly lower capacity to delay lipid oxidation than sodium nitrite.

#### 2.1.3. Saturated Fat Replacement

Fat is a main component in the production of meat products due to its importance in the sensory and microbial quality. However, meat products are important dietary sources of saturated fatty acids (SFAs) and cholesterol that have been indicated as a risk factor for the development of severe illnesses [3]. Consequently, the reformulation became necessary to produce healthier and functional meat products. The professionals of the meat industry and researchers have put emphasis on the study, production, and commercialization of healthier products, where the reduction of saturated fat is achieved [46]. Developing solutions to produce healthier products with the preservation of flavor and consumer acceptability and expectations represents an important challenge [47].

In this context, recent studies have been making efforts to improve meat product formulations with the incorporation of plant or marine oils as fat replacers, which is one of the most advantageous strategies. For instance, hazelnut oil is an interesting candidate to be a fat replacer as well as a functional food supplement, since it contains 79.05–83.16% monounsaturated fatty acids (MUFA), 7.15–9.00% saturated, and 8.47–15.66% polyunsaturated fatty acids (PUFA) [48]. Saygi, Ercoskun, and Sahin [49] studied the effects of fat substitution (15%, 30%, and 45% ratio) with hazelnut paste in traditional fermented sucuks. Among the advantages of the substitution of beef fat with hazelnut paste, cholesterol content was reduced, whereas total MUFA and PUFA proportions increased. As a disadvantage, the addition of hazelnut increased TBARS values. This result is explained by the larger proportion of unsaturated fatty acids in vegetable fats than in animal, which favors the lipid oxidation with the increasing levels of hazelnut paste replacement ratio. These researchers also found that fat substitution with hazelnut in fermented sausages significantly increased the dry matter of sausages. This outcome could be explained due to the lower moisture content of hazelnut paste than in animal fat when added to traditional sausages. Finally, although there were no significant differences in sensory scores for different samples, it is important to take notice when 2 mg/kg of TBARS is exceeded since it could be considered as a threshold for meat rancidity [50]. Therefore, after 30 days of storage and with amounts equal to or higher than 30% replacement of beef fat by hazelnut, consumers could perceive a rusty flavor. Indeed, the authors have reported that sensory scores decreased as the substitution ratio increased.

Substituting solid fat by unsaturated oils in liquid form usually affects the texture of the products. Thus, in order to obtain a novel approach to carry unsaturated oils into fermented meat formulations by compensating the lack of animal fat and preserving overall quality, several authors have investigated the utilization of gelled emulsion (GE) systems as fat replacers in fermented meat products. The GE systems are defined as emulsions with a gel-like network structure and mechanical properties similar to a viscoelastic solid that mimics the technological and sensory attributes of animal fat commonly used in meat products [51]. In this context, Alejandre et al. [30] studied different levels of animal fat replacement by a high omega-3 (ω-3) content carrageenan gelled emulsion, which was composed of linseed oil (40%), carrageenan (1.5%), and water (58.5%), in dry fermented sausages (26.3%, 32.8%, and 39.5% substitution of pork back fat). According to these authors, fat replacement did not cause relevant modifications on peroxide, TBARS, or color values. Finally, an improvement in the fatty acid composition of fermented sausages was achieved. The final acceptable product (32.8% of animal fat substitution) had 26% of fat, 2.32 g ω-3 fatty acids/100 g product, and a ω-6/ω-3 ratio of 1.87. In addition, the α-linolenic acid content increased significantly in modified sausages compared to the control (2.9 vs. 0.4 fatty acids/100 g product, respectively).

Glisic et al. [28] investigated the production of fermented sausages with inulin (I) and a GE composed of inulin and linseed oil (16%; IO) as fat replacement. The fat replacement in both treatments modified the pH of fermented sausages during ripening and the IO treatment achieved the lowest pH value. This could be explained as the result of inulin degradation by LAB. As expected, there were changes in proximate composition reflected in the reduction of total fat, a reduced content of saturated fatty acids with a favorable ω-6/ω-3 ratio in sausages with emulsion, and also a reduction in the cholesterol content. In addition, higher a_w_ values in sausages with added inulin were observed. In both modified and control sausages, a_w_ reached a value below 0.9 after 14 days of ripening. Thus, it can be considered that these reformulated products had no potential for harmful bacteria growth.

Öztürk-Kerimoğlu et al. [29] studied GE systems (peanut and linseed oils as fat replacers to replace 50% and 100% of animal fat) stabilized either with cold or hot gelation in fermented beef sausages. Among the results, it stands out that the free water content of the GE systems did not have a significant effect on a_w_ values; therefore, an equivalent drying yield was observed. The authors also indicated that sensory analysis revealed that color, texture, flavor, oiliness, rancid flavor, and overall impression were similar among treatments whereas differences were observed for visual scores and rancid flavor. The panelists reported that the rancid flavor was higher in cold-set GE samples compared to control samples, since the raw taste of the oils was transferred from the emulsion system to the fermented sausage matrix and was perceived as a rancid taste. Additionally, the authors also suggested the use of natural antioxidants and flavor enhancers to improve oxidative stability and preserve sensory quality in fermented sausages containing hot-set GE during longer storage periods.

Another strategy considered consist in the supplementation of animal feed with bioactive compounds and obtain meat with enhanced nutritional and functional properties and then it to produce healthier and functional fermented sausages. Promising results were reported for fatty acids and natural antioxidant, for instance [52,53]. To the best of our knowledge, only a single study has explored the entire chain of events connecting the supplementation of animal feed with bioactive compounds with the production of fermented sausage. Rubio et al. [54] supplemented the diet of pigs with either sunflower or soya oils (4%) and used their meat to produce “salchichón” (a traditional Spanish fermented sausage). Sausages produced with meat from animals in supplemented diet displayed increased PUFA content and reduced SFA content. Additionally, the sausages produced from PUFA enhanced meat had lower values of hardness and chewiness in texture analysis, but no major differences were reported in terms of sensory attributes (color, odor, taste, hardness, juiciness, and acceptability).

### 2.2. Adding Functional Ingredients into Meat Products

#### 2.2.1. Prebiotics

Gibson et al. [55] defined prebiotics as any substrate that is selectively used by host microorganisms in addition to those commonly present in the intestinal tract, with beneficial health results. Although most prebiotic compounds are well-established based on non-digestible carbohydrates, other substances such as polyphenols and PUFA (converted to the respective conjugated fatty acids) might be considered as a prebiotic [55]. Prebiotics are not digested in the first part of the gastrointestinal tract digestion; they find their way to the ileum and the colon, where they are digested by the gut microbes residing there. The fermentation in the lower part of the gut is characterized by the production of several end products such as the short chain fatty acids (SCFA, e.g., acetate, butyrate, and propionate) that have been closely associated with the regulation of immunological response and cancer, thus demonstrating that these microbial metabolites have several physiological effects. In this way, prebiotics could represent an interesting tool to modulate the microbiota composition and activity to the host advantage, since they can be converted by the microbiota into useful metabolites [56,57].

Due to the presence of salt, saturated fat, additives, and the low dietary fiber and bioactive compounds contents in meat products, an important product reformulation is being considered in order to use prebiotics/fibers as extenders with added nutritional value (Table 2) [58]. However, this task involves substantial knowledge in meat science and technological areas as well as sensory and regulatory aspects [55]. In this context, Park et al. [59] investigated the quality characteristics of salami sausages added with different levels (1%, 3%, and 5%) of whole buckwheat flour (BWF; rich in dietary fiber, essential minerals and vitamins B1, C, and E) during storage. The addition of BWF to salami decreased a_w_ after 21 days of storage. Additionally, the pH values of sausages decreased during storage when the added BWF contents increased, possibly due to the increment of the lactic acid production. Regarding color analysis, the redness of the salami was not affected by the introduction of BWF during storage. TBARS assay indicated an inhibition of lipid oxidation in samples treated with 3% and 5% BWF during storage. This effect could be associated with the increased free radical scavenging ability by fermentation that might result in the production of more total phenolic compounds and flavonoids in the fermented meat products. In the microbiological assessment, a selective antimicrobial activity of buckwheat was shown (5% BWF), since BWF produced a LAB increment during storage and also reduced total aerobic microorganisms.

Mikami et al. [60] supplemented dry fermented sausage with Sake lees (Sake-kasu) and studied the effects on quality characteristics over an aging period of 35 days. Sake lees are the white paste derived from the filtered residue of Japanese Sake (rice wine) mash, which is a rich source of fiber, carbohydrates, proteins, peptides, amino acids, fat, ash, vitamins, and enzymes. Supplementation with Sake lees could effectively improve the texture, smell, and safety of dry fermented sausages. On the other hand, sake lees increased acidity and produced a sour taste in dry fermented sausages, which reduced their overall acceptability. The authors concluded that the amount of Sake lees supplement should be readjusted or new formulations with flavor enhancers should be studied.

#### 2.2.2. Probiotics

Dry fermented sausages have a unique texture and typical fermentation-derived flavors. The variability in the autochthonous microbiota is one of the key aspects that dictate the characteristics and sensory attributes of traditionally fermented sausages. However, these traditionally fermented sausages generally undergo spontaneous fermentation; therefore, the quality and safety of the meat products may be affected among batches [70]. In this scenario, standardizing the production process with selected microorganisms (used as starter culture) in the traditional dried fermented sausage production is a fundamental strategy. Microorganisms isolated from traditional meat products are promising candidates for meat starter culture (usually inoculated at a rate of 10^6^ UFC/g of meat batter) since they have good adaptability to the ecological niche of fermented meats [71]. It is important to mention that safety assessment is necessary to support the selection of isolated candidates for probiotic sausages production. Applicable tests aim to characterize the presence of virulence factors, production of biogenic amines, bile salt deconjugation, hemolytic activities, and susceptibility to antibiotics. Assessment of side-effects during animal and human trials is also indicated to support the safety of probiotic candidate [72]. Modern starter cultures can be composed of coagulase-negative cocci (CNC), yeast, and LAB [73].

Nowadays, starter cultures play a role beyond quality in dry fermented sausages by providing additional health benefits. This condition is particularly relevant in the case of probiotic microorganisms. Probiotics are defined as “live microorganisms which, when administered in adequate amounts, confer a health benefit on the host” [74]. Several beneficial effects have been demonstrated from the regular consumption of probiotics, such as immune system stimulation, intestinal mucosa barrier, reduction in diarrhea occurrence, stimulation of intestinal motility, relief of lactose intolerance symptoms (due to better use of this disaccharide), production of digestive and protective enzymes, reduction of constipation, colon cancer prevention or suppression, and anti-carcinogenic effects in vivo [75,76].

The use of suitable probiotic strains in adequate doses is an important requirement. Most of the scientific evidence shows that the minimum doses from which beneficial effects are observed are between 10^6^–10^9^ UFC/g per day [77]. In order to exert its beneficial effects, it is important that the ability of the strain to survive the passage through the acidic environment of the stomach as well as the ability to adhere to the epithelium of the intestine or to the mucus layer be evaluated so that probiotic bacteria can multiply in the intestine [77]. In this context, since fermented meat sausages could represent an adverse environment for probiotic viability (due to their large amounts of salt, nitrites, low pH, and low water activity), probiotics encapsulation has emerged as an alternative to protect and improve cell viability in the case of unfavorable environments during processing and storage, as well as during gastrointestinal transit [78].

Cavalheiro et al. [61] studied the use of *E. faecium* CECT 410 as free cells or encapsulated in alginate beads on dry fermented sausages during the ripening and storage periods. The researchers found that during the ripening period (20 days), lower pH values were the consequence of a greater lactic acid accumulation as a result of *E. faecium* growth as free cells or in alginate beads. Although after 60 days of storage there were no significant differences in the probiotic count, both techniques of probiotic incorporation into the sausage could be considered successful since the counts remained above 8 log CFU/g. Thus, the authors assert that the consumption of 10 g of sausages per day at this probiotic level would be adequate to achieve the minimum recommended dosage for probiotic use.

Lately, there has been an interest in using biocompatible polymers in the microencapsulation process due to easy shaping into micrometer particles and controlled release in the human body [79]. Vasconselos et al. [62] developed functional fermented sausages by incorporating microencapsulated *Lactiplantibacillus plantarum* BG 112 in Acrycoat S100 in Milano-type salami. Acrycoat S100 is produced by methacrylic acid and methyl methacrylate co-polymerization, having been applied to microencapsulated drug protection due to its gastro resistance. As a result, Acrycoat S100 showed a pH-dependent controlled release (pH > 7.0) since the probiotic *Lactiplantibacillus* *plantarum* strain resisted the fermentation and ripening phases. Moreover, the composition, color measurement values, and sensory acceptance did not differ between the control salami produced with a commercial starter culture and the probiotic salami added with microencapsulated *Lactiplantibacillus* *plantarum* (replacing the commercial starter culture). Additionally, the LAB count was higher in probiotic salami (8 log CFU/g) than in the control (5 log CFU/g).

Pavli et al. [63] studied the effect of the *Lactiplantibacillus plantarum* L125 strain with probiotic potential on physicochemical, microbiological, and sensorial characteristics of dry fermented sausages. The authors found that the addition of *Lactiplantibacillus* *plantarum* L125 had no effect on the pH of dry fermented sausages. Regarding the sensorial assessment, differences were observed between the control and probiotic treatments, which were found in the attributes of redness, raw odor, and acidic taste. Although the acidic taste was slightly more intense in the probiotic sausages, it was not considered to be unpleasant. *Lactiplantibacillus* *plantarum* L125 showed an acceptable survival and a good competition with the starter cultures used. Additionally, adequate amounts of probiotic bacteria during the storage period at both temperatures were observed.

Many studies showed that food-grade bacteria such as lactobacilli, bifidobacterial, and propionibacteria can produce CLA isomers from linoleic acid in vitro. These CLA producing microorganisms may be used as starter cultures to increase the CLA level in fermented meat products. Özer and Kılıç [64] investigated the utilization of optimized processing conditions for high yield synthesis of conjugated linoleic acid by *Lactiplantibacillus* *plantarum* AB20–961 and *Lactiplantibacillus* *plantarum* DSM2601 in semi-dry fermented sausages. Their results indicated that CLA contents in fermented sausages were increased 21% by *Lactiplantibacillus* *plantarum* AB20–961 and 121% by *Lactiplantibacillus* *plantarum* DSM2601 after fermentation compared to the initial CLA level determined on manufacturing day. Some adverse effects on the quality of the final product were observed.

#### 2.2.3. Symbiotic

The term symbiotic defines the combination of live microorganisms with a substrate that is metabolized by host microorganisms and generate health benefits for the host [80]. This concept entails that combining prebiotic and probiotics is more effective to promote health benefits than using them separately. In symbiotic, there is an expectation that the prebiotic ingredient promotes the survival of the probiotic in the product and in the gastrointestinal tract and/or its growth in the colon [81]. However, the synergistic symbiotic phenomenon will depend on the individual’s gut microbiota composition. This phenomenon is referred to as “responders/non-responders”, representing the way in which an individual’s gut microbiota respond to prebiotic dietary interventions [82]. The intestinal human microbiota is a complex ecosystem showing important variations among individuals and it is influenced by environmental and physiological factors of the host, making its study an intricate challenge for researchers [83]. Currently, the literature on fermented meat products designed as a probiotic-prebiotic symbiotic system is still increasing. Some studies oriented to this topic are described below.

Coelho et al. [65] studied the application of *Lacticaseibacillus paracasei* LPC02 and lactulose as a potential symbiotic system in the manufacture of dry fermented sausages. Four formulations were analyzed: the control (without prebiotic or probiotic); prebiotic treatment (with 2% lactulose); probiotic treatment (with 10^8^ CFU/g of *Lacticaseibacillus paracasei*); and symbiotic treatment (with 2% lactulose and 10^8^ CFU/g of *Lacticaseibacillus paracasei*). No differences were found for the LAB count among all evaluated treatments, in any ripening time analyzed. Consequently, the authors concluded that the probiotics count and the presence of prebiotics were sufficient to consider these reformulated dry fermented sausages as potentially symbiotic. In the same line of thought, Cavalheiro et al. [84] asserted that the presence of high counts of probiotic microorganisms inoculated in fermented meat will not guarantee LAB counts larger than those found in the traditional product (without probiotic addition), being generally between 7.0 and 8.0 log CFU/g.

Similar results were found by Sirini et al. [66], who evaluated the effect of the combined use of *Lactiplantibacillus* *plantarum* and chestnut flour on Longaniza de Pascua with the hypothesis that prebiotic ingredients (chestnut flour) could promote probiotic survival in the meat product. Four formulations were analyzed: the control (without prebiotic or probiotic); prebiotic treatment (with 3% chestnut flour); probiotic treatment (with 8.5 log CFU/g of *Lactiplantibacillus* *plantarum*); and symbiotic treatment (with 3% chestnut flour and 8.5 log CFU/g of *Lactiplantibacillus* *plantarum*). However, no synergistic effect was observed between the probiotic strain and the chestnut flour in total LAB and *Lactiplantibacillus* *plantarum* counts. This could be explained by the high loads of inoculated probiotic strain (stable during fermentation), and the drying process that hindered the synergistic growth of probiotics using chestnut flour. The authors suggest other trials with lower probiotic inoculations to study this effect. Additionally, the authors found that Longaniza de Pascua could be a good alternative to carrier *Lactiplantibacillus* *plantarum* since it exhibited satisfactory sensorial scores.

Conversely, another experiment with a symbiotic pairing (fructooligosaccharides with either *Lacticaseibacillus paracasei* BGP1 or *Lactobacillus rhmanosus* GG) in low-fat dry fermented sausages showed a significant increase in the counts of LAB during processing in relation to non-inoculated and prebiotic (produced with fructooligosaccharides without probiotics) sausages [67]. In this sense, it seems reasonable to consider that the symbiotic effect in the meat product leads to increased levels of probiotic counts and may be strain-dependent. However, additional experiments are necessary to clarify the effects observed across studies of symbiotic dry fermented sausages.

Studies also indicated relevant outcomes at a human level. For example, the effect of different types of fiber (citrus fiber, arabinogalactan, and inulin), the probiotic *Lacticaseibacillus rhamnosus*, and an herbal extract in the characteristics of salami were tested by Perez Burillo et al. [68]. The antioxidant capacity, SCFA production, and gut microbiota structure of different salami formulations after in vitro digestion and subsequent fermentation with human gut microbiota was quantified. The presence of dietary fiber in the salami formulation improved the health markers of this fermented meat product since it increased sausage antioxidant capacity and the amount of SCFA produced during the microbiota fermentation. The highest values were observed in the case of citrus fiber treatments and citrus fiber with herbal extract treatments. The in vitro gut microbial fermentation test revealed that the addition of fiber to salami promoted different microbial structures compared to the traditional salami formulation by reducing the prevalence of *Escherichia*/*Shigella*. In a posterior experiment of the same research group, Perez-Burillo et al. [69] investigated the effects of ingesting 30 g/day (during 4 weeks) of salami supplemented with citrus fiber and the probiotic starter *Lacticaseibacillus rhamnosus* HN001 by 24 healthy volunteers (fecal microbiota composition, SCFA production, blood biochemistry, anthropometry, immunological markers, and antioxidant capacity of plasma and feces). The authors concluded that the reformulated salami could be used as a substitute of regular salami in common diets due to the health promoting effects in terms of inflammatory and immunological markers (CRP and TNFα), antioxidant plasmatic markers, and butyrate production. Moreover, no changes were observed in anthropometric measurements or blood biochemistry. Both studies demonstrated that the addition of fiber to fermented sausages has beneficial effects on health. In addition, the fibers studied in this food system can be considered as a prebiotic since these beneficial effects are demonstrated and occur during the fermentation of the fermented sausage by human gut microbiota in the colon.

#### 2.2.4. Polyphenols

Polyphenols are compounds with antioxidant and antimicrobial activity, which are found naturally in various plants, fruits, and vegetables. One strategy currently used is to use polyphenol-rich extracts in order to improve the shelf life of meat products [85]. Specifically, the antimicrobial activity of phenolic acids could represent a potential food additive against foodborne bacteria [86]. Phenolic acids present stronger activity at low pH, since their pKas are usually in the range of 4.5–4.8. Thus, fermented sausages could be good candidates for the use of phenolic acids due to their low pH. In this context, Meira et al. [87] investigated the combined use of essential oil compounds and phenolic acids against *Escherichia coli* O157:H7 in DFS production. The antimicrobial effect of ferulic acid (FA), *ο*-coumaric acid (CA), *ρ*-hydroxybenzoic acid (HBA), allyl isothiocyanate (AITC), and carvacrol (CAR) was determined in vitro. As a result, essential oil compounds and phenolic acids were efficient as antimicrobials at low doses in acid media, even with the resistance of *E. coli* O157:H7 in this environment. In addition, a synergism with AITC against *E. coli* O157:H7 was observed, whereas only ferulic acid was synergistic with carvacrol. On the other hand, the combined use of AITC with CA showed potential as an antimicrobial, but lower quantities of CA should be used to avoid the abrupt pH drop of the sausage batter. Thus, the authors propose future studies to investigate the AITC- CA combinations by increasing AITC up to sensorial acceptable concentrations with lower quantities of CA.

## 3. Future Healthier and Functional Fermented Meat Products: Tendencies

### 3.1. Fermented Meat Product Evolution

The development of healthier and functional fermented meat products has been centered in the direct strategies to reduce/replace sodium chloride, replace commercial nitrate and nitrite salts by natural extracts rich on nitrate or nitrite, replace saturated animal fat by vegetable oils, and add prebiotics and probiotics. Posterior experiments considered the necessity to improve the quality, safety, shelf life, and perception of healthiness of fermented sausage by consumers. Studies regarding each one of these pillars are still evolving, improving the knowledge about the broad dimension of developing healthier and functional meat products.

These initial strategies have been gradually evolving and interesting strategies and ingredients have been studied in other types of sausages and could be considered in the reformulation of dry fermented sausages. For instance, Beck et al. [88] obtained a 25% reduction in sodium content in fresh sausage with encapsulated sodium chloride in carnauba wax in order to explore the concept of taste contrast (consist in the heterogeneous distribution of an ingredient that leads to multiple temporal stimulation of taste). 

The strategies and technologies discussed in this section are characterized by the initial level of research, as well as the necessity to generate more specific knowledge, and clarify the mechanisms of action and effects in health at in vivo and a human level, for instance.

### 3.2. Residual Nitrite and Nitrosamine Accumulation and Microbial Synthesis of NO

The current trend to reduce the use of curing agents (nitrite and nitrate salts) has also required a strict control in residual nitrate and nitrite content in meat products [77]. An emerging option in this context is the use of starter cultures or prebiotics. For instance, Chen et al. [89] proposed the use of *Lactiplantibacillus plantarum* CMRC6 and *Lactobacillus sakei* CMRC15 as starters to produce Chinese fermented dry sausage. Both strains proved to reduce pH of sausage during processing, which depleted the residual nitrite content and also increase the microbiological safety. Likewise, Cheng et al. [90] obtained a 43% reduction of residual nitrite content due to the inoculation of *Lactiplantibacillus* *plantarum* GIM 1.191, *Pediococcus pentosaceus* GIM 1.428, and *Debaryomyces hansenii* GIM 2.184 in the production of Chinese Cantonese sausage. Wang et al. [91] reported a similar effect in the production of Chinese Cantonese sausage with a multi-species starter culture (*Lactobacillus sakei*, *Pediococcus pentosaceus*, *Staphylococcus xylosus*, and *Staphylococcus carnosus*) in relation to non-inoculated sausage.

Due to the key role of residual nitrite in the formation of nitrosamines, a relation between the use of starter cultures and nitrosamine formation has been suggested. This effect was observed in the study carried out by Xiao et al. [92] with *Lactobacillus pentosus* R3 inoculated in pork sausage. These authors indicated significant reduction in the formation of total nitrosamine content as well as in N-nitrosodimethylamine, N-nitrosodiethylamine, and N-nitrosopyrrolidine during processing. One of the possible explanations for the effect of starter culture in the residual nitrite content and formation of nitrosamines is the pH drop and limiting the availability of precursors (nitrite and amines) [91,92]. However, it seems that other factors may affect the relation between the use of starter cultures and residual nitrite content due to the significant differences in pH and a_w_ along with a non-significant difference in the residual nitrite content between inoculated (*Lactiplantibacillus* *plantarum* GM77 and *Staphylococcus xylosus* GM92) and non-inoculated fermented and cooked sucuk sausages [93]. Another relevant result from this study was the effect on individual nitrosamine content in fermented and cooked sucuk. The use of starter cultures caused a significant increase in the content of N-nitrosopyrrolidine and N-nitrosopiperidine, whereas no effect was reported for N-nitrosodimethylamine and N-nitrosodibutylamine content. Moreover, the increasing in the intensity of cooking (medium, medium well, well done, and very well done) was also associated with the increase in the formation of N-nitrosopyrrolidine.

The effect of starter cultures in residual nitrite content is not limited to the meat product and some evidence suggests that the effect in residual nitrite is also observed during simulated gastrointestinal digestion. Kim and Hur [94] investigated the effect of six different starter cultures on the concentration of residual nitrite in fermented sausages during in vitro human digestion. The use of *Staphylococcus carnosus*, *Pediococcus pentosaceus*, and *Pediococcus acidilactici* had the highest potential to deplete residual nitrite. Additionally, the lowest amounts of residual nitrite during simulated digestion were observed from sausages produced with *Pediococcus acidilactici* and the combination of *Pediococcus pentosaceus* and *Staphylococcus carnosus*.

Regarding the use of prebiotics, Sirini et al. [66] studied the combined use of *Lactiplantibacillus plantarum* and potential prebiotic chestnut flour in Spanish dry-cured sausage (Longaniza de Pascua). The authors reported that adding chestnut flour reduced residual nitrite and pH in Longaniza de Pascua. According to the authors, this drop in the residual nitrite levels could be explained due to the high reactivity of nitrites with the different bio-compounds present in chestnut flour, like polyphenols and flavonoids. A related experiment in mice indicated that including the inulin in fermented sausages reduced the endogenous formation of nitroso compounds during digestion [95]. According to the authors, the inulin possibly reduced the availability of nitrite or nitrate reductase produced from the gut microorganisms. Moreover, several authors reported that the real nitrite content in meat products is rather low [96,97]. Regarding residual nitrate, its presence in meat products has been detected in different meat products but is lower than levels naturally found in leafy vegetables [98].

Alternatively, the use of microorganisms with nitric oxide synthase activity has been suggested in fermented sausages without the addition of nitrate or nitrite salts [99]. In this strategy, the formation of nitric oxide is formed from the consumption of _L_-arginine from meat proteins. Additional information is necessary to increase the knowledge about the characteristics of this enzyme, its relationship with other key ingredients (such as sodium ascorbate), and the aspects related to fermented sausage (microbial safety, oxidative stability, and sensory attributes) during maturation and shelf life.

### 3.3. Postbiotics

As previously mentioned, many of the LAB are considered probiotics due to their health benefits. This consideration is related to the secretion of bioactive compounds that referred as postbiotics. The concept of postbiotics is relatively new and can be defined as a product composed of inactive microorganisms and/or their secreted metabolites that induce health benefits on the host [100]. Since the term postbiotics can be related to all components present in a microbial fermentation that induce a beneficial biological response, postbiotics can include many different constituents such as metabolites, SCFA, microbial cell fractions, bioactive peptides, exopolysaccharides (EPS), cell lysates, teichoic acid, peptidoglycan-derived muropeptides, and pili-type structures [101]. In this sense, the generation of bioactive compounds in dry fermented sausages during fermentation has been studied, especially about bioactive peptides and EPS.

#### 3.3.1. Bioactive Peptides

In the last decades, many studies have suggested that protein hydrolysis is not only attributed to the activity of endogenous meat enzymes, but it is also related to the activity of some bacterial groups, such as *Staphylococcus*. In other words, bacterial peptidases and proteases play an important role in the release of low-molecular-weight compounds, such as peptides and amino acids. For this reason, several authors have been focused on studying the effect of a possible proteolytic activity in starter cultures. Chen et al. [102] investigated the effect of a starter culture, which had been isolated from a Chinese fermented pork product (Nanx Wudl), on microbiological, biochemical, and organoleptic attributes of dry fermented sausages during processing. The starter used was the combination of *Staphylococcus xylosus* SX16 and *Lactiplantibacillus plantarum* CMRC6. The study revealed that the addition of a starter accelerated acidification and proteolysis during ripening, and also suppressed the growth of *Enterobacteriaceae* in inoculated sausages.

The microorganisms composing the starter culture play an important role in the release of bioactive peptides during sausage production, as indicated by Mejri et al. [103]. These authors explored three combinations of commercial starter cultures (*Staphylococcus xylosus* and *Lactiplantibacillus* *plantarum*, *Staphylococcus xylosus*, and *Lactobacillus pentosus*, or *Staphylococcus carnosus* and *Lactobacillus sakei*) wherein the most promising results in terms of antioxidant and antihypertensive activities were observed in sausages inoculated with *Staphylococcus xylosus* and *Lactiplantibacillus* *plantarum*.

Yu, Feng, and Sung [104] studied the influence of mixed starters (*Lactiplantibacillus plantarum* CD101 and *Staphylococcus simulans* NJ201) on protein degradation and antioxidant peptide release during dry fermented sausage processing. The results of sodium dodecyl sulfate–polyacrylamide gel electrophoresis (SDS–PAGE) analysis indicated that mixed starters could accelerate protein hydrolysis and could also delay oxidative reactions in dry fermented sausages, since the ferric ion reducing antioxidant power (FRAP) value of the peptides extracted from the starter-inoculated sausages significantly increased compared with the control treatment. A related study with *Lactiplantibacillus* *plantarum* KX881772 (alone or in combination with a commercial starter culture composed of *Pediococcus pentosaceus* and *Staphylococcus carnosus*) in dry fermented sausages revealed that this strain had relevant proteolytic activity to improve the release of peptides with antioxidant and antihypertensive activity [105]. Interestingly, this study also indicated that the type of meat (camel vs. beef) was an importance factor to favor the release of bioactive peptides wherein dry fermented sausages produced with camel meat had higher radical scavenge and angiotensin-converting enzyme inhibitory activity than beef sausage. Although promising results suggest that peptides could exert biological effects in fermented sausages, more studies are necessary to characterize the biological effects and related mechanisms of action. Additionally, more studies are necessary to clarify the impact of probiotics and their metabolites in the shelf life of fermented sausages.

#### 3.3.2. Exopolysaccharides

The EPS are biopolymers produced and secreted by microorganisms (especially LAB). These compounds are classified in homopolysaccharides (such as Alternan, Dextran, Mutan, and Reuteran) and heteropolysaccharides (Kefiran, for instance) depending on the existence of one or more types of monosaccharides in the EPS. Their effects and mechanisms of action in human organisms are not entirely known, but compiling evidence has been generated and indicates anticancer, cholesterol-lowering, antihypertensive, and antioxidant activities [106].

Regarding the developing of dry fermented sausages containing EPS, some studies indicate promising outcomes in terms of quality. Hilbig et al. [107] indicated that choice for appropriate strain (homopolysaccharides- vs. heteropolysaccharides-producing strains) plays an important role in the characteristics of the spreadable fermented sausage Teewurst. The study carried out by these authors indicated that reduced fat sausages (20% fat) produced with *Lactobacillus sakei* 1.411 and *Lactobacillus curvatus* 1.1928 (homopolysaccharide-producing strains) led to higher sensory scores from consumer than samples produced with *Lactiplantibacillus* *plantarum* 1.1478 (heteropolysaccharide-producing strain). Significant increases in pH and reductions in hardness were obtained between inoculated and non-inoculated sausages, regardless of fat content (20–40%). Interestingly, the fat content affected the production of EPS in all inoculated sausages wherein the highest contents were obtained from sausages containing 30% of fat.

Another experiment with heteropolysaccharide-producing strain *Lactiplantibacillus* *plantarum* TMW 1.1478 in salami indicated that the formation of EPS is most intense in the first 72 h of fermentation [108]. The sausages inoculated with this strain also had lower hardness and higher sensory scores for consistency (associated with soft texture). No differences in taste among treatments were observed. A related study with sucuk fermented by EPS-producing strains *Lactiplantibacillus* *plantarum* 162 R and *Leuconostoc mesenteroides* N6 (individually or combined) revealed that EPS production was incremented due to increases in ripening temperature (from 14 °C to 18 °C) and period (8–16 days) [109]. Consequently, the authors also reported that hardness, gumminess, and chewiness were also improved when temperature and time were increased. Additionally, differences in EPS production between strains were also observed wherein *Leuconostoc mesenteroides* N6 was more efficient than *Lactiplantibacillus* *plantarum* 162 R. The application of EPS-producing microorganisms in dry fermented sausages has been showing promising results, especially for sausages with soft texture and spreadable due to the effect in texture (consistency and spreadability, for instance).

### 3.4. Consumer Perception about Healthier and Functinal Meat Products

Along with the technological and health-related aspects necessary to advance the development of healthier and functional fermented sausages, the role of consumer perception and the purchase potential have to be considered in this context to connect the last stage of production chain with the broad scientific evidence generated about this topic [110]. The involvement of consumer dietary habits, preferences, and opinion is an emerging aspect related to development of healthier and functional meat products. A complex scenario is formed from studies, such as indicated in the survey carried out by Barone et al. [46], that characterize aspects such as over-processing and unfamiliarity with the new products as being negatively perceived by meat product consumers. Positive perceptions were associated with attributes such as partial replacement of meat by plant protein source and reductions in the content of salt and fat. Additionally, this study also indicated that the replacement of nitrite was positively perceived by meat products consumers. Another survey performed by Shan et al. [111] indicated a similar scenario about the uncertainty generated about the consumption of reformulated meat products. These authors indicated two main factors that could explain the lack of positive perception towards the reformulation of meat products: the first is the lack of matching between the incorporation of a healthier attribute while negatively perceived elements remain in the meat product; and the second is that healthy benefits are not easily assessed and verified, which creates the necessity to rely on a third independent part to support the health claim for consumers. Positive perceptions about the reduction of nitrite content were also reported in a survey with meat consumers [112].

Aiming for specific shares of the meat products market is another important aspect that assists in the development of healthier and functional meat products. This consideration is supported by the survey with meat consumers which revealed that fat and salt in products consumed preferentially by children are positively perceived [113]. Another outcome from this study is the positive perception of reformulation of meat products that regularly consumed. Additionally, conveying the accurate and clear message in the label has been indicate as important element to facilitate the consumer interpretation about the reformulation in the moment of purchase [46,112]. It is important to remember that the information discussed in this section involves a broad perspective about the reformulation of meat products, and specific knowledge about dry fermented sausages requires additional studies to strengthen guidance.

### 3.5. Recommendations

The development of healthier and functional meat products also conveys the microbiological safety requirement. This condition is an important concern in terms of sodium chloride and nitrate and nitrite salts. Although important advances have been reported for sodium chloride, the same progression has not been reported in the development of reducing nitrate and nitrite salts. The complete removal of nitrate and nitrite salts has not been properly solved yet due to its key importance in multiple characteristics of fermented sausages, especially in microbiological safety. Therefore, strategies to reduce nitrate and nitrite salts are necessary in future experiments, but their complete removal without a proper substitute is not a decision that meat producers would be willing to take without ensuring the same level of quality and safety of the current commercial fermented sausages.

Considering the increasing importance and scientific information from consumer studies, it is relevant to consider that reformulation should join the healthier and functional products in order to align scientific advances with consumer preferences. In this sense, the information presented in the label must be presented in a clear and accurate manner in order to effectively communicate the modifications in the reformulated meat product. Current legislation applied in Europe covers part of the health claims (such as for salt and fat reduction) [114] discussed in this review that can be considered in future studies about the development of reformulated meat products. 

## 4. Conclusions

The development of healthier and functional fermented sausages has been evolving in the last decades. The initial and most fundamental changes in the key ingredients are associated with an increased risk of developing diseases and also with the incorporation of prebiotics and probiotics. The study of healthier and functional fermented sausages is evolving to consider more intricate and comprehensive concerns about residual nitrite, nitrosamine accumulation, postbiotics, and consumer preference. These emerging topics can be considered of great value to promote de-diversification of healthier and functional meat products. Therefore, a promising scenario lays ahead of healthier and functional dry-cured sausages development.

## Figures and Tables

**Table 1:** Strategies for the reformulation of dry fermented sausages.

Type of Fermented Sausage	Reformulation	Recommendation	Ref.
Chinese dry sausage	Sodium chloride reduction	2% and 4% NaCl	[22]
Dry fermented sausages		NaCl reduction from 2.5% to 1.0%	[24]
Dry fermented sausages	Sodium chloride replacement	Substitution of 30% of NaCl with KCl and flavor enhancers	[25]
Dry fermented sausages	Nitrate–rich extract addition	Beetroot and radish powders	[26]
Dry fermented sausages	Natural extract addition	Grape seed extract and olive pomace hydroxytyrosol and chestnut extract with olive pomace hydroxytyrosol	[27]
Fermented sausages	Fat replacement	Inulin gelled suspension and inulin linseed oil gelled emulsion	[28]
Fermented beef sausages		Gelled emulsion systems of peanut and linseed oils	[29]
Dry fermented sausages		Gelled emulsion of high omega-3 and carrageenan content	[30]

**Table 2:** Fermented sausages produced with prebiotics, probiotics, and symbiotics.

Fermented Sausage	Category	Functional Ingredient	Ref.
Salami	Prebiotics	Whole buckwheat flour	[59]
Dry fermented sausages		Sake lees (Sake-kasu)	[60]
Dry fermented sausages	Probiotic	*Enterococcus faecium* CECT 410 as free cells or encapsulated in alginate beads	[61]
Milano-type salami		Microencapsulated *Lactiplantibacillus* *plantarum* BG 112 in Acrycoat S100	[62]
Dry fermented sausages		*Lactiplantibacillus plantarum* L125	[63]
Semi-dry fermented sausage		*Lactiplantibacillus plantarum* AB20–961 and *Lactiplantibacillus plantarum* DSM2601	[64]
Dry fermented sausage	Symbiotic	*Lacticaseibacillusparacasei* LPC02 and lactulose	[65]
Longaniza de Pascua		*Lactiplantibacillus plantarum* and chestnut flour	[66]
Dry fermented sausage		*Lacticaseibacillusparacasei* BGP1 or *Lactobacillus rhmanosus* GG with fructooligosaccharides	[67]
Salami		*Lacticaseibacillus rhamnosus* with different fibers (citrus fiber, arabinogalactan, and inulin)	[68]
Salami		*Lacticaseibacillus rhamnosus* HN001 with citrus fiber	[69]

## Data Availability

Not applicable.
